# Glycemia and Atherosclerotic Cardiovascular Disease: Exploring the Gap Between Risk Marker and Risk Factor

**DOI:** 10.3389/fcvm.2020.00100

**Published:** 2020-06-09

**Authors:** Avital Nahmias, Priska Stahel, Changting Xiao, Gary F. Lewis

**Affiliations:** Division of Endocrinology, Department of Medicine, Banting & Best Diabetes Centre, University of Toronto, Toronto, ON, Canada

**Keywords:** cardiovascular risk, diabetes mellitus, glycemia, atherosclerosis, macrovascular complications

## Abstract

There is consistent, unequivocal and reproducible epidemiological evidence derived from diverse populations that various indices of glycemia (fasting plasma glucose, post-prandial or post oral glucose challenge plasma glucose, HbA1c) are associated with an increased risk of atherosclerotic cardiovascular disease (ASCVD), even in the prediabetic state. Furthermore, there is abundant experimental evidence demonstrating that hyperglycemia *per se* accelerates and aggravates the atherosclerotic process, providing biological plausibility to the concept that hyperglycemia is causally related or a true risk factor for ASCVD. Two studies in particular, DCCT and UKPDS, that enrolled a younger cohort of patients with type 1 diabetes or an older cohort with newly diagnosed type 2 diabetes, respectively, showed trends toward a reduction in ASCVD. The reductions in ASCVD reached statistical significance only after prolonged follow up, and when differences in HbA1c were no longer maintained (referred to by some as a “legacy effect”). More recent studies in those with established type 2 diabetes, in which glycemic control was improved by a variety of strategies, failed to demonstrate reductions in ASCVD. The gap in evidence supporting hyperglycemia as a true causative risk factor for ASCVD or simply a risk marker for some other confounding causative factor is discussed in this review. We conclude that hyperglycemia does appear to be at least partially causative of ASCVD (i.e., an ASCVD risk factor). We discuss how this evidence can be incorporated into an overall therapeutic strategy to prevent ASCVD in those with prediabetes and established diabetes.

## Introduction

Atherosclerotic cardiovascular disease (ASCVD) is the leading cause of morbidity and mortality in people living with diabetes. Advances in the prevention and management of ASCVD have reduced mortality (1). Epidemiological studies have consistently supported a strong association between various indices of glycemia (fasting plasma glucose, post-prandial or post oral glucose challenge plasma glucose, HbA1c) and risk of ASCVD. However, there is still much debate as to whether hyperglycemia should be regarded as a risk marker (i.e., associated but not causative) or a true risk factor (i.e., a causative factor for ASCVD). Both risk factors and risk markers are associated with increased risk, however risk factors are defined by the ability to reduce the risk by correction of the variable. Although intuitively, in view of the tight association between glycemia and ASCVD and biological plausibility based on experimental evidence, one might assume that improved glycemic control would reduce the risk of ASCVD, most studies have failed to demonstrate a robust reduction in ASCVD with improved glycemic control. Long-term follow-up of glycemic control studies have provided new insights that shed additional light on the relationship between glycemia and ASCVD. This brief review does not provide a comprehensive review of all available evidence but instead highlights key evidence to illustrate important relationships. First, we will briefly review cross-sectional population data and experimental evidence of pathophysiological links, the latter providing biological plausibility for hyperglycemia as a causative factor for ASCVD. Next, we will discuss the results of the major, prospective, randomized, controlled, clinical trials of the treatment of hyperglycemia by various modalities that have for the most part failed to demonstrate a robust reduction in ASCVD as the primary study outcome. Based on this evidence we will address the gap that exists between hyperglycemia as a risk marker vs. risk factor for ASCVD and will speculate on the factors that may explain this discrepancy.

## Glycemia as a Consistent and Powerful Risk Marker for ASCVD—Results From Cross-Sectional Population Studies

Numerous studies in those with diabetes have demonstrated unequivocally that hyperglycemia is a potent risk marker for ASCVD (2, 3). Studies have also demonstrated that hyperglycemia in the non-diabetic range is associated with ASCVD (4). In the Glucose Tolerance in Acute Myocardial Infarction study, glucose intolerance was the single most powerful predictor of cardiovascular complications and death (5). Similar results were reported in the Asian Pacific study, showing that higher fasting plasma glucose levels correlate with increased risk for stroke and ischemic heart disease (IHD) (6). In addition to the fasting or peak glucose levels affecting ASCVD risk, a higher variability of glucose levels was shown to correlate with carotid intima media thickness (7) and with oxidative stress (8). Similarly, post-prandial glucose has also been associated with a higher CVD risk (9–11).

In the Multiple Risk Factor Intervention trial, regardless of any other ASCVD risk factors, patients with diabetes had an odds ratio of 2–4 for ASCVD mortality, in comparison to patients without diabetes (12). This was further validated in the European Prospective Investigation of Cancer and Nutrition (EPIC Norfolk) (13) and in the Atherosclerosis Risk in Communities (ARIC) analysis (14). In summary, this non-exhaustive list of studies that have linked hyperglycemia, even in the non- or pre-diabetic range, to increased risk for ASCVD, provide convincing evidence that hyperglycemia is a powerful risk marker for ASCVD.

## Potential Pathological Mechanisms of Hyperglycemia—Building a Case for Biological plausibility

Numerous studies have examined potential pathophysiological mechanisms whereby hyperglycemia may be directly or indirectly implicated in accelerating the atherosclerotic process. A number of reviews in this issue of Frontiers in Cardiovascular Medicine and others have discussed various aspects of this topic in detail (15). Here we will briefly highlight the most prominent mechanisms that have been described.

Prolonged hyperglycemia is observed both in type 1 and type 2 diabetes. Endothelial cells have limited ability to regulate glucose influx, leaving them more vulnerable to high intracellular glucose concentrations, excessive reactive oxygen species (ROS) production and oxidative stress. Excessive ROS production is believed to play a key role in the cellular dysfunction observed in the person with diabetes, linked to the many processes that promote atherosclerosis, cardio-toxicity and insulin resistance (16). ROS promotes the generation of Methylglyoxal, the major contributor to generation of a heterogenous group of chemical moieties known as advanced glycation end products (AGEs). AGEs contribute to endothelial dysfunction, vasoconstriction and pro-inflammatory and pro-atherogenic changes, that underlie atherosclerotic plaque rupture and thrombus formation (17). Increase glucose flux divert glucose from glycolysis to alternative pathways such as polyol and hexosamine pathways, exacerbating glycation and protein kinase C activation (18, 19). ROS have been shown to activate PKC isoforms, again contributing to endothelial dysfunction (20).

High intra-cellular Ca2+ levels, induced by ROS activate NFAT (nuclear factor of activated T cells). NFAT activation has been linked to accelerated atherosclerosis, cardiac toxicity and inflammation (21). The pro-inflammatory milieu promotes atherosclerosis and plaque rupture (22), however it also disrupts pancreatic beta cell function, aggravating hyperglycemia (23), which in turn generates oxidative stress that provokes an inflammatory response (24–26), forming a vicious cycle. Finally, hyperglycemia has also been shown to promote activity of 12- and 15-lipoxygenase, enzymes that react with fatty acids and generate lipid metabolites that are pro-atherogenic (19).

While prolonged hyperglycemia is observed in both type 1 and 2 diabetes, type 2 diabetes caries marked insulin resistance and hyperinsulinemia, with further deleterious effects. Insulin resistance is associated with an increase in free fatty acid (FFA), further contributing to ROS production (27). Additionally, insulin resistance decreases the myocardium ability to utilize glucose, due to a decrease in glucose transporter 4 (GLUT4) expression (28). Together, it makes the myocardium dependent on fatty acid as a source of energy, generating more cardiac toxicity (25).

The above-mentioned intracellular pathways interfere with key endothelial functions, including loss of nitric oxide vasodilatory effect, increased expression of inflammatory cytokines and adhesion molecules (29, 30). An increase in endothelial cell permeability allows the infiltration of monocytes into the intimal layer, influx of lipoproteins and the ultimate generation of foam cells, the basis of the fatty streak and eventually the atherogenic plaque. People with diabetes consistently have an atherogenic plaque characterized by a higher lipid concentration and more inflammatory cells (31), making the plaque more prone to rupture and thrombus formation.

In addition to vascular injury, patients with diabetes also present a pro-coagulable state, with a decrease in the fibrinolytic pathway and heightened platelet activity (32). Hyperinsulinemia and hyperglycemia have been linked to higher levels of plasminogen activator inhibitor-1 (PAI-1), factor VII, XII and fibrinogen and lower levels of tissue plasminogen activator (tPA), favoring a pro-coagulant state (33). In addition, chronic hyperglycemia causes an increase in glycoprotein IIb-IIIa and P-selectin expression on the platelet surface, and an increase in the sensitivity of PY212, an activating platelet receptor, all promoting platelet activation and thrombus formation (34).

Furthermore, neurologic injury that may result from hyperglycemia *per se*, could potentially impair the sensation of myocardial ischemic symptoms in those with diabetes, leading to late diagnosis and treatment, which further contributes to the ASCVD morbidity and mortality. In summary, numerous pathophysiological mechanisms have been described that provide biological plausibility to the notion that hyperglycemia can directly promote atherosclerosis and worsen the outcome of cardiovascular events.

## The DCCT and UKPDS Trials Failed to Demonstrate Significant Reductions in ASCVD but did Demonstrate a “Legacy” Effect After Prolonged Follow up (Table 1)

The Diabetes Control and Complications Trial (DCCT), which enrolled relatively young adults with type 1 diabetes (T1D), demonstrated a non-significant trend toward reduced CV events with improved glycemic control (average HbA1c in the intensively insulin-treated group was 7% and that in the usual care group was 9%). Of note, the relatively young age of the DCCT study participants was reflected in a low overall incidence of ASCVD (0.5 in the treated patients vs. 0.8 event per 100 patient-years in the control group), thus making the study underpowered to demonstrate a reduction in ASCVD (40). With prolonged follow up of an additional 9 years following unblinding of the main study results (EDIC study), a significant reduction in ASCVD events was demonstrated in the original intensive insulin treatment group (41, 42), despite the fact that the initial difference in glycemic control was not maintained during follow up. Similarly, the initial analysis of the UK Prospective Diabetes Study (UKPDS) trial of newly diagnosed individuals with T2D failed to demonstrate that more aggressive vs. standard glycemic control using a variety of glucose lowering therapeutic modalities significantly reduced ASCVD (*p* = 0.052), while a clear reduction in microvascular complications was observed (39). The average HbA1c in the intensively treated UKPDS cohort was 7% and that in the usual care cohort was 7.9%, with HbA1c rising with time in parallel in both cohorts. Metformin treatment in obese patients in the UKPDS study was shown to reduce the incidence of myocardial infarction (MI) by 39% (*p* = 0.01). However, the metformin-treated cohort was relatively small and the results would need to be replicated in a larger study population to confidently confirm that metformin is antiatherogenic (43). Following completion and unblinding of the UKPDS study population, a long-term follow-up study (UKPDSFU) continued to follow some of the study population with no specific intervention in terms of glycemic control. With a follow-up of 10 years, a significant reduction of 15% in the incidence of MI and 17% reduction in mortality from diabetic complications was demonstrated in the original intensively treated group, despite the fact that the original study difference in glycemic control became non-significant 1 year after termination of the initial study (44). These results of the UKPDSFU and the EDIC study pointed to a legacy effect of early glycemic control that became evident 10 years after the initial study ended, despite no ongoing difference in treatment between the groups during this follow up time period (42). A study with a follow-up of 18 years showed a similar reduction in ASCVD related mortality both in patients with type 1 and type 2 diabetes, age adjusted HR of 5.2 and 4.9, respectively (45). There has been much speculation regarding the mechanism of the legacy effect. Hyperglycemia for prolonged periods has been shown to generate an inflammatory and fibrotic gene signature in endothelial and smooth muscle cells, with evidence of specific epigenetic changes. Interested readers are referred to a number of excellent reviews on the topic (46–48). Of note, as discussed in the next section, a similar “legacy effect” has not been replicated in other clinical trials.

**Table 1:** Effects on CV outcomes of landmark randomized controlled trials with the goal of intensifying glycemic control.

	**ADVANCE (35)**	**ACCORD (36, 37)**	**VADT (38)**	**UKPDS (39)**	**DCCT (40, 41)**
Number of patients	11,140	10,251	1,791	5,102	1,441
Mean Age (years)	66	62.2	60.4	53.3	26.9
nitial BMI	28	32	31.3	27.5	23.5
HbA_1c_ Achieved (%) Intensive vs. standard	6.5 vs.7.3	6.4 vs.7.5	6.9 vs. 8.4	7 vs. 7.7	7 vs. 9
Mean FU (years)	5	3.5	5.6	10	6.5
CV outcome	MACE: - Non-fatal MI - Non-fatal stroke - CV death	MACE: - Non-fatal MI - Non-fatal stroke - CV death	Composite CV events: - MI - Stroke - CV death - CHF - Inoperable CAD - Surgical intervention for CVD - Amputation for ischemic gangrene	Myocardial Infarction	MACE: - Non-fatal MI - Non-fatal stroke - CV death
Duration and severity of diabetes	Vascular disease or risk factor 8 years of diabetes	CVD or 2 risk factors m 10 years of diabetes	Poorly controlled 11.5 years of diabetes	New onset type 2 diabetes	5.9 years of type 1 diabetes
Risk reduction for CV outcome	*HR* = 0.94 (0.84–1.06) *P* = 0.32	HR = 0.9 (1.04–0.78) P = 0.16	*HR* = 0.87 (0.73–1.04) *P* = 0.14	*RR* = 0.84 (0.71–1) *P* = 0.053	NS
Glucose lowering drugs	Gliclazide, metformin, thiazolidinediones, acarbose, or insulin	Metformin, sulfonylureas, meglitinides, thiazolidinediones, α-glucosidase inhibitors, insulin, and exenatide	Glimepiride, metformin, rosiglitazon, and insulin	Chlorpropamide, glibenclamide, glipizide, metformin, and insulin	Insulin pump or injections

*CV, cardiovascular; RR, Relative Risk; HR, hazard ratio; FU, follow up; CHF, congestive heart failure; CI, Confidence Interval; MI, myocardial infarction; BL, baseline; NS, Non significant; MACE, Major adverse cardiovascular events; CAD, Coronary artery disease*.

## Improvements in Glycemia in Three Landmark Clinical Trials That Demonstrated no Significant Beneficial Effect in Preventing ASCVD (Table 1)

The above-mentioned studies prompted studies that examined the effect of even more stringent glycemic control to near-normal levels and whether the benefit of glycemic control was limited to newly diagnosed patients with diabetes. Three major studies compared stringent glycemic control in patients with T2D: the Action to Control Cardiovascular Risk in Diabetes (ACCORD) study (36), the Action in Diabetes and Vascular Disease: Preterax and Diamicron Modified Release Controlled Evaluation (ADVANCE) study (35) and the Veterans Affairs Diabetes Trial (VADT) study (38). All three studies achieved a significant and sustained difference in glycemia between intensive and standard treatment arms: in the ACCORD trial: HbA1c 6.4 vs. 7.5%, the ADVANCE: HbA1c 6.4 vs. 7.0%, the VADT: HbA1c 6.9 vs. 8.5%. All three studies failed to demonstrate a significant benefit on ASCVD risk, with minor trends toward reduction (6–13%) in CV events, that did not reach statistical significance (35, 38, 49, 50). Moreover, the ACCORD study was terminated after 3.5 years due to a 22% increase in overall mortality, demonstrating a possible harmful effect of intensive glycemic control (49). As mentioned above, in contrast to the “legacy effect” demonstrated in the DCCT and UKPDS studies, prolonged follow-up of these studies failed to demonstrate a similar legacy effect. In the VADT study the trend toward reduced risk for ASCVD disappeared as the difference in glycemic control became insignificant (51). A 6-year post trial follow-up of the ADVANCE trial, showed no difference in the risk of death from any cause or major macrovascular events, between the intensive- and standard glucose control groups (52). In the ACCORD trail, a 17 months follow-up after the early termination, also failed to demonstrate a significant difference in major cardio-vascular events between the groups (53). A longer follow up of 9 years did show an increase in cardiovascular-related death, but no change in all-cause death and non-fatal cardiovascular events (54).

## Explaining the Gap Between Glycemia as a Risk Marker vs. Risk Factor

Several explanations for the absence of robust cardiovascular benefit of improved glycemic control in the ACCORD, ADVANCE and VADT trials have been suggested, however none has been validated in a study designed to test these hypotheses. Therefore, we can only speculate on the mechanisms of the gap between risk marker and risk factor.

The first, obvious potential speculation is that hyperglycemia is not directly atherogenic in humans (despite *in vitro* and animal experimental findings) but instead is associated with a confounding factor that is the causative factor accelerating atherosclerosis. There are many potential candidate factors that could be operative in persons affected by diabetes. To name a few; hyperlipidemia, hypertension, chronic inflammation, prothrombotic state, microalbuminuria or overt nephropathy, and endothelial dysfunction. The obesity, insulin resistance and hyperinsulinemia of Type 2 diabetes may also be implicated.

Secondly, the long standing hyperglycemia in the ACCORD, ADVANCE and VADT trials might have caused diffuse and irreversible atherosclerosis and cardiovascular injury, which were beyond the beneficial effect of later improvements in glycemic control. This is again in contrast to the study population of the UKPDS and DCCT that recruited younger patients in the DCCT and newly diagnosed patients in the UKPDS. Evidence supporting this hypothesis was found where patients with a higher level of coronary atherosclerosis measured by coronary-artery calcium score showed a lesser reduction in ASCVD events (55). The proatherosclerotic changes that may occur as a direct result of hyperglycemia may not be reversible by glucose lowering after a prolonged duration of hyperglycemia. Along these lines, subgroup analysis of the studies mentioned above demonstrated that intensive treatment benefited predominantly the younger and newly diagnosed population, similar to the cohort of the UKPDS and DCCT trials (41, 43).

Another hypothesis is that cardiovascular events triggered by severe hypoglycemia may offset any potential cardiovascular benefits that may occur with intensive glycemic control. Hypoglycemia was hypothesized but not proven to play a role in the excess mortality observed in the ACCORD trial. This was mainly due to the low target HbA1C (<6) which was suspected as a cause for excess hypoglycemia events, and the association between hypoglycemia and triggering of cardiac events (56). However, in a later analysis of the ACCORD data, aimed to better identify the population(s) at higher risk of mortality, only three significant interactions were found between baseline characteristics and effects of intensive vs. standard glycemia treatment on mortality: self-reported history of neuropathy, higher HbA1c and aspirin use (36). Furthermore, the risk of death appeared to be greater with the intensive compared to the standard strategy only when the average HbA1c was >7% (57). Obesity and polypharmacy with potential drug interactions were also suggested as possible explanations of the increased mortality.

It is feasible that cardiovascular benefits may only be seen when improvements from very poor to improved glycemic control are achieved but not when improving those who have milder degrees of hyperglycemia. Although the DCCT and UKPDS trials did not demonstrate significant reductions in ASCVD outcomes during the trials, they did demonstrate reductions after prolonged follow up.

Another possible cause for the lack of benefit was the aggressive treatment of other risk factors with statins, aspirin and ACE inhibitors in the more recently conducted ACCORD, ADVANCE, and VADT trials. Reduced mortality and morbidity to below what is expected in patients with T2D might have diminished the added effect of aggressive glycemic control. This was further evident in the STENO-2 trial. Intensified multifactorial intervention with tight glycemic control and the use of ACE inhibitors, aspirin, and lipid-lowering agents has been shown to reduce ASCVD events by 59%, CV related mortality by 57%, and all-cause mortality by 46% (58). Of note, the UKPDS and DCCT studies were performed before the widespread use of statins and ACE inhibitors, again suggesting that the beneficial effect of tight glycemic control is less evident when other risk factors such as hypertension and hypercholesterolemia are treated.

## Conclusions

There is a gap between hyperglycemia as a consistent, reproducible risk marker demonstrated in numerous epidemiological studies and the somewhat underwhelming evidence of reduction in ASCVD in glucose lowering intervention trials. When considering all available evidence, hyperglycemia does appear to be at least partially causative (i.e., an ASCVD risk factor). Reductions in ASCVD have been demonstrated in patients with more recently diagnosed T2D or in younger patients with T1D. This is in keeping with the abundant experimental evidence that has demonstrated biological pathways in which hyperglycemia *per se* can accelerate the atherosclerotic process. The benefit is relatively small, however, and takes many years to manifest, in contrast to the more rapid and robust cardiovascular benefits of other therapies such as LDL lowering, antihypertensive therapy, inhibition of the renin angiotensin system and two of the newer classes of glucose lowering therapies, the SGLT2 inhibitors and GLP-1 receptor agonists. Given the major benefits of glucose lowering in reducing diabetic microvascular complications and the added small benefit of glucose lowering in reducing macrovascular events, optimization of glycemic control remains the cornerstone of diabetes therapy, especially in the context of microvascular complications. One should take heed, however, of the risks of aggressive control of hyperglycemia that have been well-demonstrated in numerous clinical trials, particularly that of severe hypoglycemia. As lowering glucose levels appears not to be the most effective measure to reduce ASCVD complications and aggressive control has not resulted in a clear benefit in those with long standing diabetes and milder levels of hyperglycemia and even might result in excess mortality, practicing physicians must carefully balance benefits vs. risks, as has been aptly highlighted in all national diabetes guidelines. Finally, as macrovascular complications start to develop well before clinically evident diabetes is diagnosed, non-glucose and modifiable ASCVD risk factors such as hypercholesterolemia, hypertension and smoking cessation should be aggressively implemented in the pre-diabetic phase.

## Author Contributions

AN wrote the manuscript. PS and CX critically reviewed the manuscript. GL wrote and reviewed the manuscript.

## Conflict of Interest

The authors declare that the research was conducted in the absence of any commercial or financial relationships that could be construed as a potential conflict of interest.
